# Antimicrobial Peptides and Their Therapeutic Potential for Bacterial Skin Infections and Wounds

**DOI:** 10.3389/fphar.2018.00281

**Published:** 2018-03-28

**Authors:** Anja Pfalzgraff, Klaus Brandenburg, Günther Weindl

**Affiliations:** ^1^Pharmacology and Toxicology, Department of Biology, Chemistry, Pharmacy, Institute of Pharmacy, Freie Universität Berlin, Berlin, Germany; ^2^Brandenburg Antiinfektiva GmbH, Borstel, Germany

**Keywords:** antimicrobial peptides, topical therapy, skin and soft tissue infections, wounds, wound healing, bacterial resistance, biofilms, bacterial toxins

## Abstract

Alarming data about increasing resistance to conventional antibiotics are reported, while at the same time the development of new antibiotics is stagnating. Skin and soft tissue infections (SSTIs) are mainly caused by the so called ESKAPE pathogens (*Enterococcus faecium, Staphylococcus aureus, Klebsiella pneumoniae, Acinetobacter baumannii, Pseudomonas aeruginosa*, and *Enterobacter* species) which belong to the most recalcitrant bacteria and are resistant to almost all common antibiotics. *S. aureus* and *P. aeruginosa* are the most frequent pathogens isolated from chronic wounds and increasing resistance to topical antibiotics has become a major issue. Therefore, new treatment options are urgently needed. In recent years, research focused on the development of synthetic antimicrobial peptides (AMPs) with lower toxicity and improved activity compared to their endogenous counterparts. AMPs appear to be promising therapeutic options for the treatment of SSTIs and wounds as they show a broad spectrum of antimicrobial activity, low resistance rates and display pivotal immunomodulatory as well as wound healing promoting activities such as induction of cell migration and proliferation and angiogenesis. In this review, we evaluate the potential of AMPs for the treatment of bacterial SSTIs and wounds and provide an overview of the mechanisms of actions of AMPs that contribute to combat skin infections and to improve wound healing. Bacteria growing in biofilms are more resistant to conventional antibiotics than their planktonic counterparts due to limited biofilm penetration and distinct metabolic and physiological functions, and often result in chronification of infections and wounds. Thus, we further discuss the feasibility of AMPs as anti-biofilm agents. Finally, we highlight perspectives for future therapies and which issues remain to bring AMPs successfully to the market.

## Introduction

Antimicrobial peptides (AMPs) are increasingly coming into the focus as new treatment strategies for bacterial infections. But why new options have to be considered? The golden era of antibiotic discovery that started almost one century ago when Fleming discovered penicillin in 1928 and that can be accounted as one of the greatest achievements of modern medicine seems to be over. While up to the 1980s it was popular opinion that infections have been conquered (Spellberg et al., 2004, 2008), today alarming data about increasing resistance to conventional antibiotics are reported occurring due to excessive use and misuse in medicine, food industry, and agriculture. The current resistance situation raises concerns about a post-antibiotic era with no antimicrobial treatment options. At the same time, the development of new anti-infective drugs is stagnating with only three new classes of antibiotics during the last decades—all of them exclusively directed against Gram-positive pathogens (Marr et al., 2006). Estimates claim that by 2050 worldwide 10 million people a year will die from infections caused by drug-resistant bacteria (Cordes et al., 2014) and the World Health Organization classifies the appearance of antibiotic resistance as one of the biggest threats to human health.^1^

The so called ESKAPE pathogens (*Enterococcus faecium, Staphylococcus aureus, Klebsiella pneumoniae, Acinetobacter baumannii, Pseudomonas aeruginosa*, and *Enterobacter* species), representing the most recalcitrant bacteria, are resistant to almost all common antibiotics and are the leading causes of hospital-acquired infections (Santajit and Indrawattana, 2016). As skin and soft tissue infections (SSTIs) are mainly caused by *S. aureus*, with MRSA (methicillin-resistant *S. aureus*) accounting for 50% of all SSTIs, but also Gram-negative pathogens such as *P. aeruginosa* and vancomycin-resistant *Enterococcus* (VRE) are increasingly isolated, especially from chronic wounds, the current resistance situation constitutes a major issue for the treatment of these infections (Guillamet and Kollef, 2016). Moreover, SSTIs rank among the most common bacterial infections in humans (Sunderkotter and Becker, 2015) and increasing resistance is reported against topical antibiotics such as fusidic acid or mupirocin (Mendoza and Tyring, 2010; McNeil et al., 2011; Guillamet and Kollef, 2016). Thus, new treatment options for bacterial SSTIs are urgently needed. Although SSTIs can be provoked by other microbes such as viruses, fungi and parasites, this review focuses on bacterial skin infections since bacteria play a major role for skin infections in humans.

AMPs, which are already known for several decades and are part of the innate immunity from practically all living organisms, ranging from bacteria, insects and plants to vertebrates, are supposed to be promising candidates for the treatment of skin infections and wounds. Their discovery started in 1939 with the extraction of gramcidin from *Bacillus brevis* followed by the isolation of cecropins and magainins in the 1980s (Kang et al., 2017). During the last years substantial progress has been made on the development and evaluation of AMPs for the treatment of skin infections as well as acute and chronic wounds. The purpose of this review is to discuss the suitability of AMPs as potential therapeutics to treat bacterial skin infections and wounds. Therefore, we will introduce the different opportunities given by the application of AMPs for topical infections and wounds, highlight their advantages and limitations and try to contribute to answering the question if AMPs are indeed promising candidates for the treatment of bacterial SSTIs and wounds and which issues remain to introduce them successfully to the market.

## Antimicrobial barrier function of the skin

**Figure 1:**
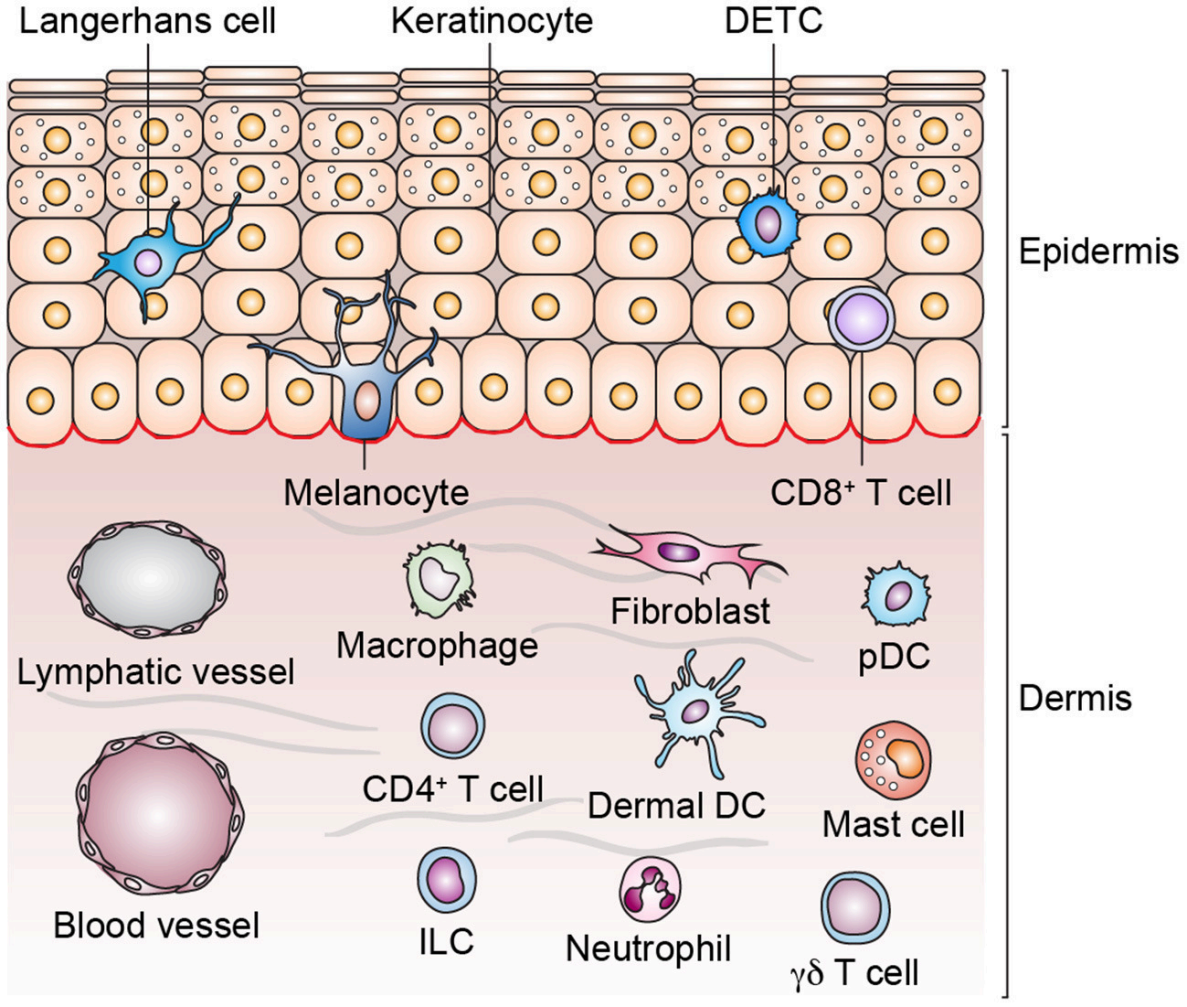
Structure of human skin and cell types in epidermis and dermis. Skin appendages are not depicted and the list of cell types is non-exhaustive. Dendritic epidermal T cell (DETC), dermal dendritic cell (dermal DC), innate lymphoid cell (ILC), plasmacytoid dendritic cell (pDC).

The skin is our outermost barrier and thus exposed to a considerable number of diverse pathogens. Consequently, it has to provide a vast arsenal of defense mechanisms. Apart from the physical barrier that is preventing the penetration of microbial organisms and mainly provided by the stratum corneum, the outermost layer of the skin, the epidermis harbors an imposing number of immune sentinels that are activated by invading pathogens or barrier disruption (Bangert et al., 2011; Ryu et al., 2014). A generalized structure of human skin and different cell types populating the skin is shown in Figure 1.

Keratinocytes, the predominant cell type of the epidermis, which do not only maintain the physical barrier, but are central sentinels of the skin, greatly contribute to the defense against bacterial skin infections. They express various Toll-like receptors (TLRs) and nucleotide-binding oligomerization domain 2 (NOD2) proteins which are essential for recognizing pathogen-associated molecular patterns (PAMPs). Recognition of PAMPs results in the activation of innate immunity involving secretion of various cytokines and chemokines but also AMPs which enable immune cells to be recruited to the site of infection (Nestle et al., 2009; Krishna and Miller, 2012a). While activation of TLRs in the skin is required for activation of innate immunity and subsequent adaptive responses and thus for resolution of infections, excessive stimulation can lead to overwhelming inflammatory responses that might cause inflammatory skin diseases, autoimmune diseases, or even sepsis (Lai and Gallo, 2008). In addition to keratinocytes, TLRs are expressed in dermal fibroblasts and Langerhans cells located in the epidermis, but also in other resident and trafficking immune cells such as monocytes, macrophages and dendritic cells, lymphocytes, and mast cells. Skin-residing cells show distinct TLR expression patterns and can therefore recognize distinct PAMPs to initiate different host defense mechanisms (Miller, 2008).

## Physiological role of AMPs in the skin

A pivotal function of skin innate immunity is implemented by AMPs. More than 20 AMPs have been identified in the skin (Takahashi and Gallo, 2017). Since AMPs do not only possess direct antimicrobial effects but are likewise able to enhance and control immune responses as will be reviewed later, they are able to strongly support the immune response triggered by bacteria. All human body sites that are exposed to microbes such as skin and mucosal epithelia are abundant with AMPs to prevent colonization of host tissues by pathogens.

Endogenous AMPs are derived from immature pro-peptides that have to be cleaved proteolytically to obtain biologically active mature peptides (Hemshekhar et al., 2016). The best characterized AMPs in humans—the cathelicidin LL-37 and the defensins—are produced by various cell types, including keratinocytes, macrophages, neutrophils, mast cells and dendritic cells (Krishna and Miller, 2012b; Afshar and Gallo, 2013). To generate LL-37, the parent cathelicidin molecule hCAP18 has to be processed via proteinase 3 in neutophils and via kallikreins in keratinocytes (Takahashi and Gallo, 2017). The most important source for AMPs in the skin are keratinocytes that constitutively express AMPs such as human β-defensin 1 (hBD1), while others like hBD2-4 are induced when exposed to injury or infection. The expression of hBD2 for example can be induced by bacterial components targeting TLR2 or TLR4. LL-37 is constitutively expressed in neutrophils, but can also be induced by vitamin D3 (VD3) or lipopolysaccharide (LPS) in keratinocytes (Bernard and Gallo, 2011). The subsequently recruited neutrophils and mast cells are likewise producing AMPs resulting in a strong AMP increase which is leading to the stimulation of angiogenesis and proliferation of keratinocytes to repair the injured tissue (Lee et al., 2005; Afshar and Gallo, 2013). α-defensins (human neutrophil peptides—HNPs) are highly expressed in neutrophils which account for almost 50% of neutrophil granules (Ryu et al., 2014). Furthermore, the cationic peptide RNase7 is produced in keratinocytes, while the anionic peptide dermcidin is produced by human eccrine sweat glands (Bangert et al., 2011). The AMP psoriasin was found to protect the skin against *E. coli* infection (Glaser et al., 2005). AMPs are also produced by commensal bacteria such as phenol-soluble modulin (PSM-)γ which is expressed in *Staphylococcus epidermidis* (Takahashi and Gallo, 2017) and displays direct antimicrobial activity against *S. aureus* and indirect by activating TLR2 on keratinocytes, thereby enhancing the production of endogenous AMPs (Ryu et al., 2014). Mast cells were only recently recognized to play a role for immune defense against skin infections by producing cathelicidin peptides which recruit neutrophils and show a direct antimicrobial effect and thus protect from GAS (group A *streptococcus*) skin infection and help to prevent the extension of localized Gram-positive skin infections (Di Nardo et al., 2008). On the other side, the expression of cathelicidins in mast cells contributes to skin inflammation in rosacea (Muto et al., 2014) demonstrating the dual role of AMPs.

## AMPs and skin diseases

**Figure 2:**
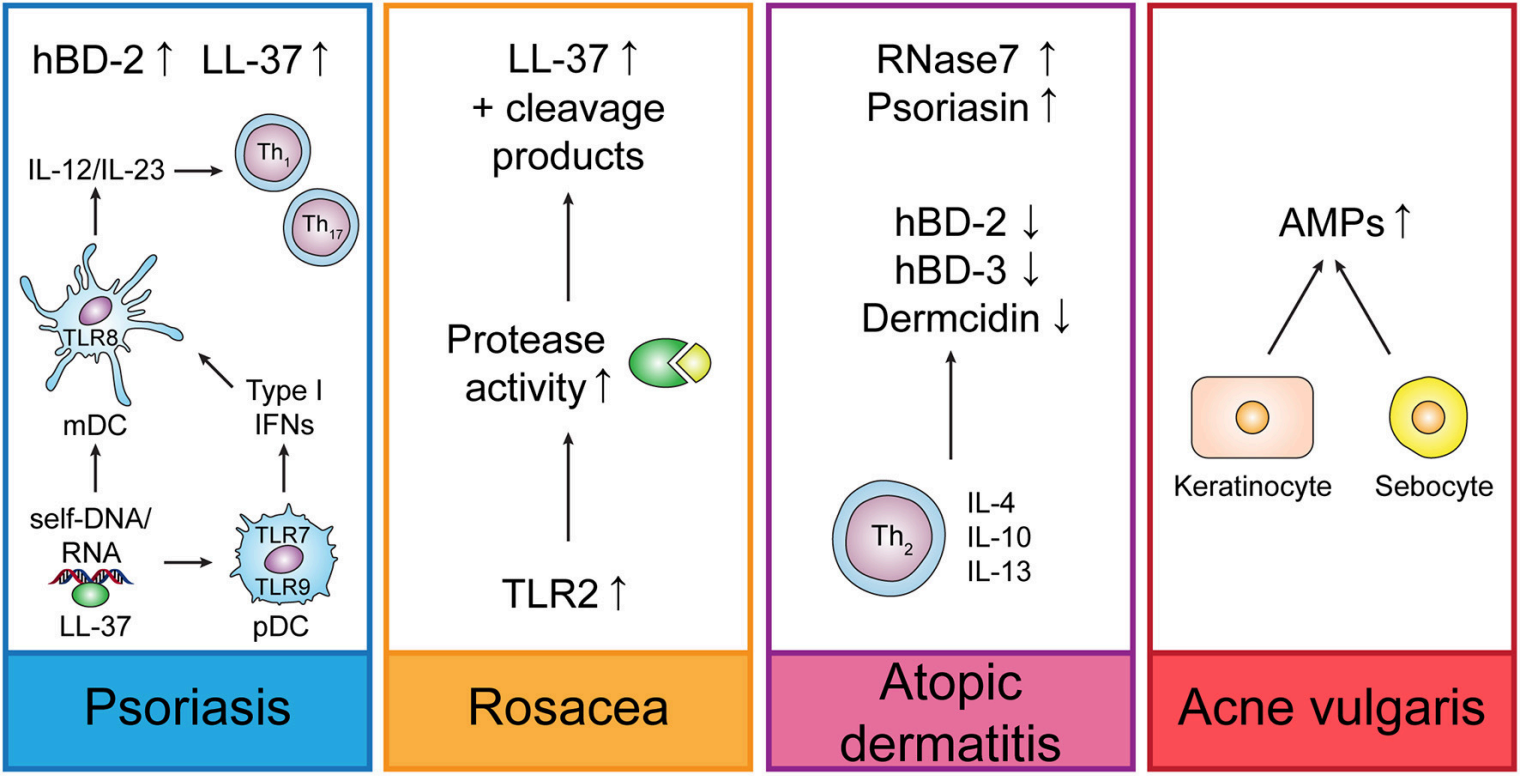
Regulation of endogenous AMPs in different skin diseases. In psoriasis, LL-37 is forming complexes with self-DNA or -RNA, thus leading to the activation of plasmacytoid dendritic cells (pDCs) and myeloid dendritic cells (mDCs) which trigger Th_1_ and Th_17_ responses by secretion of IL-12 and IL-23. Rosacea is characterized by increased TLR2 expression which triggers LL-37 production and increases protease activity leading to unusual LL-37 cleavage products. In atopic dermatitis, Th_2_-derived cytokines suppress the induction of AMPs. In acne vulgaris, *C. acnes* (formerly *P. acnes*) induces up-regulation of AMPs in keratinocytes and sebocytes.

The function of AMPs in the skin is outlined by their role in skin diseases such as psoriasis or atopic dermatitis (Figure 2). While AMPs are downregulated in skin lesions of atopic dermatitis patients, thus predisposing patients to *S. aureus* skin infections, they are upregulated in psoriasis and rosacea where they worsen the inflammation that correlates with disease severity.

Mice with disrupted *Cnlp* (gene coding for CRAMP) show an increased susceptibility to skin infections (Ramos et al., 2011) and the deficiency of LL-37 in chronic ulcers—probably due to bacterial proteases—may influence their healing process. In contrast, hBD-2, 3, and 4 are overexpressed in diabetic foot ulcer, but not sufficient to control wound healing and infections (Rivas-Santiago et al., 2012).

In psoriasis, production of LL-37 and hBD-2 is increased, whereas LL-37 is able to form complexes with self-RNA resulting in the activation of TLR7 in plasmacytoid dendritic cells (pDCs) and TLR8 in myeloid DCs and to enable pDCs to recognize self-DNA through TLR9 and therefore contributes to psoriasis pathogenesis by IFN-γ-induced activation of myeloid dendritic cells (mDCs) and keratinocytes and Th_1_/Th_17_ differentiation (Bangert et al., 2011; Takahashi and Gallo, 2017). Likewise, hBD2, hBD3, and lysozyme were shown to induce pDC activation in psoriasis (Lande et al., 2015).

Rosacea is accompanied by up-regulation of TLR2 which in turn triggers cathelicidin production leading to abnormally high levels in the epidermis and unusual cathelicidin cleaving products (Dutta and Das, 2015). A role of LL-37 in the pathogenesis of rosacea could be demonstrated by the application of ivermectin in keratinocytes and 3D skin models which displayed an anti-inflammatory effect by suppressing expression of LL-37 and the protease kallikrein-related peptidase (KLK)5 (Thibaut de Menonville et al., 2017).

Cathelicidins, hBD-2 and 3 and dermcidin are down-regulated, while RNase7 and psoriasin are up-regulated in atopic dermatitis (AD) lesions (Takahashi and Gallo, 2017). Down-regulation of AMPs in atopic dermatitis might be supported by the presence of Th_2_-derived cytokines IL-4, IL-10, and IL-13 which suppress the induction of AMPs. However, AMPs can also play a pathogenic role in AD by stimulating the production of Th_2_-derived cytokines which are involved in AD pathogenesis (Niyonsaba et al., 2017). On the other side, it could be demonstrated that hBD-3 improves the skin barrier function which is impaired in AD (Kiatsurayanon et al., 2014).

In acne vulgaris, various AMPs are upregulated in keratinocytes and sebocytes induced by *Cutibacterium* (formerly *Propionibacterium*) *acnes* which might worsen the inflammatory response due to pro-inflammatory effects displayed by the AMPs or might be beneficial due to anti-inflammatory and antimicrobial effects (Korting et al., 2012; Niyonsaba et al., 2017).

## Structure and mode of action of AMPs

Antimicrobial peptides are evolutionarily conserved components of the innate immune system from almost all species of life ranging from plants and insects to complex animals. Bacteriocins with lantibiotics as the most popular group are produced by bacteria and used as food preservatives (Ben Lagha et al., 2017). Insect AMPs are one of the largest groups of known antibiotics and can be detected in insect haemolymph as early as 2–4 h after a septic injury (Hoffmann et al., 1993; Meister et al., 1997). Defensins are an important part of the innate immunity in plants and show strong antifungal activity (Parisi et al., 2018). Additionally, several AMPs have been isolated from frog skin and show potential activity against skin infections (Ladram and Nicolas, 2016; Liu et al., 2017). More than 2,800 natural peptides^2^ are currently known that, despite being of variable length, sequence, and structure, share pivotal characteristics that are decisive for their mode of action. The structure of representative natural and synthetic AMPs is shown in Table 1. For details on structure and mechanism of action of AMPs we refer to other recent reviews (Jenssen et al., 2006; Nguyen et al., 2011; Wang, 2014).

**Table 1:** Primary sequences of selected natural and synthetic antimicrobial peptides with wound-healing activities.

**Peptides**	**Primary amino acid sequence**
AH90	ATAWDFGPHGLLPIRPIRIRPLCG
Catestatin	SSMKLSFRARAYGFRGPGPQL
CW49	APFRMGICTTN
DRGN1	PSKKTKPVKPKKVA
Epi-1	GFIFHIIKGLFHAGKMIHGLV
Esculentin-1a(1-21)NH_2_	GIFSKLAGKKIKNLLISGLKG
LL-37	LLGDFFRKSKEKIGKEFKRIVQRIKDFLRNLVPRTES
Melittin	GIGAVLKVLTTGLPALISWIKRKRQQ
Pep19-2.5	GCKKYRRFRWKFKGKFWFWG
Pep19-4LF	GKKYRRFRWKFKGKLFLFG
SHAP1	APKAMKLLKKLLKLQKKGI
SR-0379	MLKLIFLHRLKRMRKRLkRK
Tiger17	WCKPKPKPRCH
Tylotoin	KCVRQNNKRVCK
WRL3	WLRAFRRLVRRLARGLRR

*Amino acid sequences are given in one-letter code. Lowercase letters indicate D-amino acid residues*.

Most AMPs are small (12–50 amino acids) and have a positive net charge (+2 to +9) and ~50% hydrophobic residues enabling them to fold into an amphiphilic confirmation when they interact with bacterial membranes. This structure is essential for their mechanism of action as initial electrostatic interaction between the cationic peptides and the anionic constituents of the bacterial membrane is not sufficient for their antimicrobial action. Rather insertion into the bacterial lipid membrane in a second step is required resulting in transmembrane pore forming, formation of ion channels, or membrane disruption (Kaconis et al., 2011; Mangoni et al., 2015).

AMPs are more or less selective for bacterial membranes since mammalian membranes are enriched with zwitterionic rather than with negatively charged phospolipids and possess cholesterol which protects the membrane from damaging effects by reducing the activity of AMPs stabilizing the lipid bilayer or by directly interacting and neutralizing them (Giuliani et al., 2007).

Two classes of AMPs mainly targeting bacterial membranes are described: membrane disruptive and non-membrane disruptive AMPs with intracellular targets which may act independently or synergistically with membrane permeabilization (Giuliani et al., 2007). For the pore formation induced by membrane disruptive peptides different models are suggested such as the barrel-stave (e.g., for alamethicin) or toroidal-pore model (e.g., magainin II, melittin or LL-37), carpet model (e.g., polymyxin B, dermaseptin, cecropins) (Falanga et al., 2016) and aggregate model (e.g., indolicidin) (Giuliani et al., 2007) where cell death occurs owing to metabolite leakage, cell lysis, or dissipation of electrochemical ion gradients from the bacterial cell membrane. The direct antimicrobial effect, however, occurs not only due to membrane rupture, but also extends to interference with enzymes, cell wall synthesis, nucleic-acid synthesis, and protein synthesis when non-membrane disruptive peptides translocate across the bacterial cellular or the nuclear membranes to access intracellular targets (Brogden, 2005; Ageitos et al., 2017). For example, buforin II inhibits DNA and RNA synthesis, while PR-39 decreases protein synthesis and phyrrhocidin and apidaecin target the ATPase activity of DnaK resulting in the accumulation of misfolded proteins and thus cell death.

Most AMPs show rapid and broad-spectrum antimicrobial properties (Jenssen et al., 2006; Brogden and Brogden, 2011; Mojsoska and Jenssen, 2015). However, several AMPs possess multiple targets and can simultaneously target the membrane and intracellular molecules such as NK-18, a truncated peptide derived from the core region of NK-lysin, which is membrane-active, but can also interact with plasmid and genomic DNA from *E. coli* (Yan et al., 2013).

Comparable to the antibacterial activity of AMPs, antifungal effects are mainly based on two distinct mode of actions. Membrane traversing peptides can lead to pore formation or have specific fungal targets such as β-glucan or chitin synthesis, while non-membrane traversing peptides interact with the cell membrane and cause cell lysis (Bondaryk et al., 2017). Porcine myeloid antimicrobial peptide-36-derived peptides showed antifungal activity against *Candida albicans* via membrane disruption (Lyu et al., 2016) and a synthetic multivalent antifungal peptide displayed higher antifungal activities against *Candida* and *Fusarium* strains than azoles or polyene antifungals by selectively damaging the fungal membrane (Lakshminarayanan et al., 2014). Histatin 5 was internalized by *C. albicans* and acted on mitochondrial membranes, whereas its activity was dependent on the energy status of the fungal cell (Ruissen et al., 2001). In contrast, antifungal activity of the cathelicidins LL-37 and CATH-2 did not depend on the cellular energy-status, but they rapidly localized to the cell membrane, whereas at sub-MFCs small amounts of the peptides were internalized, suggesting additional intracellular targets (Ordonez et al., 2014). Moreover, naturally produced cathelicidins are present at fungal infection sites and might form a barrier to *C. albicans* infection (Lopez-Garcia et al., 2005).

Some AMPs display strong antiviral activity by integration into the viral envelope or host cell membrane (Lakshmaiah Narayana and Chen, 2015). They can bind to heparan sulfate moieties on the cell surface and thus prevent infection with distinct enveloped viruses including herpes simplex virus (HSV)-1 and -2 (Krepstakies et al., 2012), or disrupt the envelope and/or capsid of adenovirus and HSV-1 (Gordon et al., 2009). LL-37 was able to enhance the antiviral activity induced by double-stranded RNA in keratinocytes and was potent against influenza virus A, probably by disrupting the viral membrane, and LL-37 expression in keratinocytes and B cells reduced the viral load of varicella zoster virus (Takiguchi et al., 2014; Wang et al., 2014).

AMPs can also be active against parasites, whereas most studies focus on their activity against *Plasmodium* and *Leishmania* (Vizioli and Salzet, 2002). Oocyst development in *Plasmodium* spp. was reduced by cecropin and magainin (Gwadz et al., 1989) and insect defensins were highly toxic to isolated sporozoites *in vitro* (Shahabuddin et al., 1998), while dermaseptin killed *Leishmania* by altering the permeability of the plasma membrane (Hernandez et al., 1992).

Beyond direct antimicrobial activity, AMPs also may inhibit the strong inflammation induction caused by the bacterial PAMPs (pathogenicity factors, also called toxins), essentially lipopolysaccharides (LPS, endotoxins) for Gram-negative and lipoproteins, -peptides (LP) for Gram-positive strains (Brandenburg et al., 2016). The findings in various studies that peptidoglycans and lipoteichoic acids are also relevant toxins, could not be verified (Martinez de Tejada et al., 2015), because the inflammation signals induced by LP were by at least two orders of magnitude higher that those induced by the latter compounds.

Besides the indicated antimicrobial and anti-toxin activity, AMPs may also have immunomodulatory functions which are often physiologically more relevant. AMPs may facilitate clearance of pathogens by modulating cellular immune responses, for example by reducing the levels of pro-inflammatory cytokines, modulating the expression of chemokines and reactive oxygen (ROS) and nitrogen (RNS) species, stimulating angiogenesis, enhancing wound healing, and activate and/or differentiate leukocytes and macrophages (Hilchie et al., 2013). As example, it was reported, that the particular peptide IDR-1, a 13-mer, was successful in protecting mice against Gram-negative and Gram-positive bacteremia, and at the same time, the innate immune system was stimulated by the peptide by increasing the expression of cytokines and chemokines (Scott et al., 2007). Here and in other investigations of the immune-modulating activity of peptides, however, it was not analyzed whether the compounds show any binding affinity to the bacterial toxins which are responsible for the heavy inflammation. Therefore, despite the described encouraging animal experiments, the application of immunomodulating compounds so far did not result in an improvement of the situation of critically ill patients (Opal et al., 2014).

On the basis of their secondary structure AMPs can be classified into four major categories: α-helical like the human cathelicidin LL-37 (Zeth and Sancho-Vaello, 2017), β-sheet with α-, β-, und θ-defensins as representatives, loop (stabilized by a disulfide bridge, e.g., esculentin-1; Mangoni et al., 2015) and extended peptides which are rich in particular amino acids such as tryptophane in indolicidin.

## Structural requirements for the antimicrobial activity

A number of different structural requirements are essential to obtain strong antimicrobial activity of AMPs. For synthetic peptides derived from the human cationic peptide ubiquicidin a correlation between the isoelectric point value and the antimicrobial activity against MRSA *in vitro* and *in vivo* could be demonstrated (Brouwer et al., 2006). Furthermore, the highest antimicrobial activity toward MRSA was achieved with synthetic fragments containing an α-helix, while peptides containing a β-sheet only displayed poor activity, probably due to low lipophilicity and linear charge density. The extent and ratio of cationicity, hydrophobicity, α-helicity, and amphipathicity of AMPs are decisive for the antimicrobial and cytolytic activity. For the frog skin peptide kassinatuerin-1 and its L- and D-lysine-substituted derivates increasing cationicity with maintaining the amphipathic α-helical character enhanced the antimicrobial potency against Gram-positive bacteria, but also hemolytic and cytolytic activity. On the contrary, analogs with D-amino acids retained activity against Gram-negative bacteria, while their hemolytic and cytolytic activity was reduced. However, due to the D-amino acid substitution the α-helicity and thus potency against Gram-positive bacteria were decreased.

In general, an increase in positive charge facilitates the interaction of AMPs with the bacterial cell wall, but increases hemolytic activity (Conlon et al., 2005). Also reducing the hydrophobicity of AMPs can reduce hemolytic activity (Giuliani et al., 2007). Another group investigated the effect of D-amino acids that were introduced into non-cell-selective α-helical AMPs on cell selectivity and protease-stability, depending on the number and distribution of D-amino acids introduced (Wang et al., 2010). Increase of D-amino acids and dispersed distribution rather than segregated distribution of the D-amino acids caused increase in cell selectivity toward bacterial cells. Furthermore, introduction of D-amino acids caused complete resistance to tryptic digestion. Increasing hydrophobicity and α-helicity of these peptides increased their hemolytic and anti-inflammatory activity. The short synthetic peptides RRIKA and RR showed potent antimicrobial activity against clinical and drug-resistant *S. aureus* isolates with stronger effects when amphipathicity, hydrophobicity, and net charge were increased (Mohamed et al., 2014). Bacterial killing appeared due to pore formation and disruption of the bacterial membrane leading to leakage of cytoplasmic contents and cell lysis, but also DNA binding could be demonstrated for the peptides. Importantly, RRIKA was still active in the presence of 10% fetal bovine serum and both peptides retained their antibacterial activity in the presence of increasing salt concentrations.

## Common bacterial skin infections

Skin and soft tissue infections (SSTIs) rank among the most common bacterial infections in humans and bacteria accountable for SSTIs show increasing resistance against commonly used antibiotics (Eckmann and Dryden, 2010; Amara et al., 2013). Bacterial SSTIs can range from superficial, often self-limiting mild infections without requirement for antibiotic treatment to complicated infections that result in the development of sepsis with lethal outcome. Different classifications for SSTI are proposed. A classification frequently used in clinical trials which is provided by the FDA differentiates between uncomplicated and complicated SSTI, while for the latter major surgical intervention might be necessary or deep soft tissue is involved with signs of systemic sepsis (Eckmann and Dryden, 2010). Systemic infection and involvement of deeper tissues is largely mediated by toxins that are produced by bacteria and by the host response to infection. Especially for complicated SSTIs, an epidemiological shift toward drug-resistant bacteria can be observed. *S. aureus* as the major cause of nosocomial wound infections is secreting toxins, virulence factors and exoproteins resulting in delayed wound healing, prolonged inflammation and chronic infection. Additionally, skin infections caused by *S. aureus* or *P. aeruginosa* frequently lead to invasive infections that might result in sepsis (Thangamani et al., 2015; Guillamet and Kollef, 2016). While *S. aureus* is the most common cause of SSTIs worldwide, followed by β-hemolytic *streptococci, E. coli*, and *P. aeruginosa*, chronic or postoperative wounds are predominantly caused by Gram-negative bacteria such as *P. aeruginosa, Enterococcus*, and *Acinetobacter* species (Cardona and Wilson, 2015; Esposito et al., 2016; Guillamet and Kollef, 2016). Furthermore, community-acquired MRSA is increasing which causes 60–80% of staphylococcal skin infections in the US (Stein and Wells, 2010). Notably, it could be observed that the inflammatory response after a bacterial infection contributes to the clinical severity of *S. aureus* skin infections rather than the bacterial burden (Mohamed et al., 2014).

## Current challenges of conventional anti-infectives and significance of AMPs for the treatment of SSTIs

After pneumonia and abdominal infections, SSTIs are the third most cause of severe sepsis or septic shock (Eckmann and Dryden, 2010). Conventional antibiotics, especially those targeting the bacterial cell wall, might display the major drawback of triggering the release of bacterial pathogenicity factors and therefore may even worsen the outcome of an infection by triggering sepsis or septic shock, as demonstrated for the antibiotic ciprofloxacin (Brandenburg et al., 2011; Heinbockel et al., 2013; Brandenburg and Schürholz, 2015). Chronic wounds such as diabetic wounds are often accompanied by excessive release of pro-inflammatory cytokines, thus remaining in the inflammatory phase of wound healing and can also be accompanied with insufficient angiogenesis (Liu et al., 2014a). Here, the mode of action of AMPs that neutralize bacterial pathogenicity factors or control and balance the host immune response rather than acting directly on bacteria, might be favorable to improve the outcome of infections; on the one side by enhancing levels of immune cells and chemokines that are clearing infections and on the other side by decreasing pathogenicity factor-induced secretion of pro-inflammmatory cytokines (Mansour et al., 2015; Brandenburg et al., 2016).

Antimicrobial peptides display major advantages compared to conventional antibiotics. Besides their broad-spectrum activity against various strains of Gram-positive and Gram-negative bacteria, including drug resistant strains, they show a different mode of action which is not involving specific protein binding sites or can involve multiple bacterial cellular targets making resistance development rather unlikely (Marr et al., 2006; Wimley and Hristova, 2011; Seo et al., 2012). Importantly, they successfully retained their antimicrobial activity for millions of years. Furthermore, only their direct antimicrobial effect is affected by bacterial resistance, whereas their immunomodulatory properties stay unaffected (Stein and Wells, 2010). Particularly SSTIs are an attractive target for the application of AMPs since topical treatment circumvents potential systemic adverse effects and can achieve high drug concentrations at the target site.

On the other side, AMPs possess some limitations that hamper their clinical and commercial development such as high production costs, potential toxicity, susceptibility to proteases (also in the wound fluid), and unknown pharmacokinetics (Dutta and Das, 2015). Nevertheless, various approaches are already offered including insertion of non-natural or D-amino acids, introduction of peptide-mimetics, peptide cyclisation, acetylation, or amidation at the N-terminus to avoid peptide degradation (Giuliani et al., 2007) or the design of short peptides to reduce production costs. Also the use of drug delivery systems like liposome encapsulation might help to improve their stability and reduce their toxicity (Seo et al., 2012). Therefore, several synthetic AMPs were produced that show promising effects *in vivo* after topical application on infected skin with lower toxicity than AMPs used in clinical practice such as colistin and with the advantage to not select for resistant mutants in bacterial cultures (Brunetti et al., 2016).

## Resistance to AMPs

**Figure 3:**
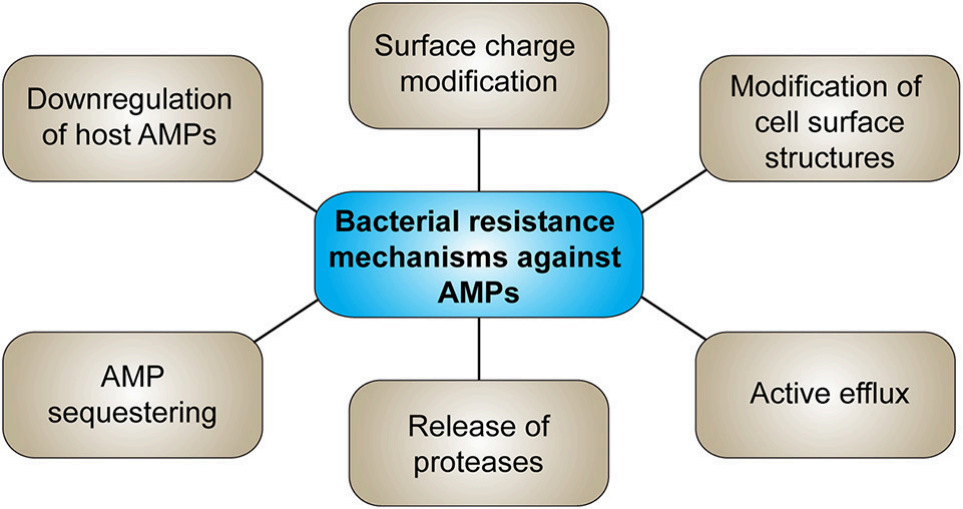
Possible bacterial resistance mechanisms against AMPs.

Bacterial resistance against AMPs has already been reported (Ouhara et al., 2008; Andersson et al., 2016; Omardien et al., 2016) holding the risk that bacteria that evolve resistance against externally applied AMPs might develop cross-resistance to host AMPs or antibiotic therapy (Dobson et al., 2014; Kubicek-Sutherland et al., 2017), such as *S. aureus* which developed resistance to HNP-1 after treatment with pexiganan (Conlon, 2015). However, synthetic peptides which do not appear in nature, could overcome the obstacle of resistance to natural host defense peptides (HDPs) (Nijnik and Hancock, 2009). Figure 3 summarizes the main bacterial resistance mechanisms against AMPs. Resistance of bacteria to AMPs might develop due to changing of their net surface charge, whereas mainly modification of LPS in Gram-negative and lipoteichoic acid in Gram-positive bacteria by increasing positive charges reduces the attraction of AMPs (Joo et al., 2016). Further, release of proteases and therefore proteolytic degradation of the peptides is a potential resistance mechanism. Commensal bacteria on epithelial surfaces produce various proteases such as metalloproteases or serine endopeptidases. Inactivation of AMPs by bacterial proteases strongly depends on the peptide structure, given a higher susceptibility to degradation of linear peptides compared to cyclic peptides containing disulfide bonds. Modifications of the bacterial phospholipid composition and thus alteration of the cytoplasmic membrane structure such as amino acylation of phosphatidylglycerol head groups, which masks anionic phosphates with cationic primary amines, decreases AMP attraction and membrane insertion. Also efflux pumps belonging to the RND (resistance-nodulation-cell division) family transporters in Gram-negative bacteria contribute to AMP resistance. Sequestering of AMPs, e.g., of HNP-1 and -2 by *S. aureus* staphylokinase, or down-regulation of host AMP production by interference with or suppression of AMP production further protects bacteria from AMPs (Andersson et al., 2016; Cole and Nizet, 2016; Joo et al., 2016). Resistance to AMPs can further reduce susceptibility to antibiotics (Kubicek-Sutherland et al., 2017). Resistance mechanisms that contribute to virulence *in vivo* are modification of cell surface structures and efflux transporters whereas only few studies demonstrated a contribution of proteases to AMP resistance *in vivo* (Bauer and Shafer, 2015).

## AMPs for the treatment of SSTIs

### Wound healing

The epidermis provides a protective barrier against microbial pathogens. However, this barrier can be disrupted by wounding, thus allowing microbial pathogens to invade the underlying tissue. After wounding, a complex wound healing process is initiated which comprises three phases. The inflammatory phase is dominated by blood clot formation and invasion of immune cells such as neutrophils and macrophages, the second phase comprises tissue formation with angiogenesis and re-epithelialization and the third phase includes remodeling with collagen synthesis (Werner and Grose, 2003). Adequate wound closure is a prerequisite for maintaining skin homeostasis since failures during wound recovery can cause the formation of chronic wounds which often remain in the inflammatory stage and are challenging to heal (Portou et al., 2015). Chronic wounds like diabetic, pressure and venous leg ulcers are mainly polymicrobial and most frequently caused by *S. aureus* and *P. aeruginosa*. While *S. aureus* is usually located in the top layer of the skin, *P. aeruginosa* is detected in the deepest region of the wound bed (Serra et al., 2015). Infection of wounds can lead to bacteremia or sepsis and is accompanied by high mortality and morbidity (Hirsch et al., 2009).

Various studies reveal the importance of natural AMPs in wound healing which are upregulated in all stages of wound recovery (Niyonsaba et al., 2017). LL-37 is upregulated in skin wounds and LL-37 antibodies suppress re-epithelialization, while in chronic ulcers LL-37 expression is low (Heilborn et al., 2003). On the contrary, adenovirus-mediated LL-37 gene transfer to excisional wounds in obese mice improved re-epithelialization and granulation tissue formation (Carretero et al., 2008). Most wounds are contaminated by bacteria and colonization of wounds can result in delayed or impaired wound healing. For treatment of infected wounds AMPs should display low cytotoxicity and broad-spectrum antimicrobial activity, they should promote wound closure and be stable in the host environment with high salt concentrations and in the presence of proteases secreted by the host, e.g., in the wound fluid, or by invading microbes in the wound site (Kim et al., 2014). In the wound fluid more than 100 endogenous proteases are identified such as metalloproteases and neutrophil elastase, but also proteases from bacteria that contaminate wounds such as *S. aureus* V8 proteinase. The synthetic peptide SHAP1 showed high stability in the presence of proteases and high salt concentrations and therefore was able to promote wound healing *in vivo* at the same low concentrations used *in vitro* (Kim et al., 2014). Degradation by proteases might be less relevant for AMPs showing rapid killing of bacteria. The designer peptide novispirin G10 killed bacteria within 4 h after intradermal injection into a *P. aeruginosa*-infected, partial-thickness burn wound before it was inactivated by proteases (Steinstraesser et al., 2002). Furthermore, rapid bactericidal activity of AMPs shows the advantages over conventional antibiotics to reduce the treatment duration and the potential for resistance development (Mohamed et al., 2016). On the other side, for peptides such as LL-37 which are unstable in the presence of proteases, much higher concentrations will be required to obtain a therapeutic effect *in vivo*. A recent study showed that omiganan probably failed clinical trials due to the degradation by skin proteases which could be overcome by using the all-D enantiomer (Ng et al., 2017). AMPs display various mechanisms that support wound healing of infected as well as non-infected wounds that will be summarized below. Table 2 provides an overview of different endogenous and synthetic AMPs and their role in different stages of wound healing. For some AMPs wound closure in mice was only accelerated in infected, but not in aseptic wounds (Huang et al., 2014), probably by facilitating wound recovery due to combating infection, whereas synthetic and recombinant LL-37 supported wound regeneration in a sterile wound model in dexamethasone-treated mice by increasing vascularization and re-epithelialization (Ramos et al., 2011). Hence, different mechanisms of AMPs affect distinct steps of the wound healing process. While AMPs with immunomodulatory and antimicrobial properties might be used to treat infected wounds, an influence on migration and proliferation of skin cells is essential to support healing of non-infected wounds.

**Table 2:** Role of selected natural and synthetic antimicrobial peptides during wound-healing phases.

**Peptides**	**Inflammatory phase (Phase 1)**	**Tissue formation phase with angiogenesis and re-epithelialization (Phase 2)**	**Remodeling phase with collagen synthesis (Phase 3)**	**References**
AH90	TGF-β ↑ in macrophages via NF-κB and JNK	Keratinocyte migration, re-epithelialization, granulation tissue formation, up-regulation of integrins	TGF-β ↑ → α-SMA ↑ → fibroblast-to-myofibroblast transition	Liu et al., 2014b
Catestatin		Keratinocyte migration and proliferation via GPCRs, PLC, EGFR, Akt/PI3K, MAPK		Hoq et al., 2011
CW49	Macrophage recruitment and pro-inflammatory cytokines ↓ in diabetic wounds	HUVEC tube formation, up-regulation of pro-angiogenic proteins		Liu et al., 2014a
DRGN1		Granulation tissue formation, re-epithelialization, keratinocyte migration and proliferation via EGFR and STAT3		Chung et al., 2017
Epi-1		Keratinocyte migration and proliferation	Formation of collagen	Huang et al., 2017
Esculentin-1a(1-21)NH_2_		Keratinocyte migration via EGFR and STAT3		Di Grazia et al., 2015b
LL-37		Keratinocyte migration via ADAM-mediated EGFR transactivation, HUVEC proliferation and formation of vessel-like structures via FPRL1		Koczulla et al., 2003; Tokumaru et al., 2005
Melittin		Keratinocyte migration via purinergic receptor activation and ADAM-mediated EGFR transactivation		Sommer et al., 2012
Pep19-2.5, Pep19-4LF		Keratinocyte migration via purinergic receptor activation and metalloprotease-mediated EGFR transactivation		Pfalzgraff et al., 2016
SHAP1		Keratinocyte migration via EGFR transactivation and STAT3, re-epithelialization		Kim et al., 2014
SPINK9		Keratinocyte migration via purinergic receptor activation and ADAM-mediated EGFR transactivation		Sperrhacke et al., 2014
SR-0379		Granulation tissue formation, HUVEC proliferation and tube formation, keratinocyte migration, fibroblast proliferation via PI3K/Akt/mTOR	Collagen production, fibroblast-collagen matrix contraction	Tomioka et al., 2014
Tiger17	TGF-β ↑ in macrohages via JNK; TGF-β ↑ and IL-6 ↑*in vivo*; macrophage recruitment	Keratinocyte migration and proliferation, fibroblast proliferation, re-epithelialization	α-SMA ↑	Tang et al., 2014
Tylotoin	TGF-β ↑ and IL-6 ↑ in macrophages (via ERK and JNK); TGF-β ↑*in vivo*; recruitment of macrophages	Keratinocyte migration and proliferation, fibroblast proliferation, HUVEC proliferation and tube formation, re-epithelialization, granulation tissue formation	α-SMA ↑	Mu et al., 2014
WRL3		Re-epithelialization, VEGF production → formation of new blood vessels		Ma et al., 2017

### Treatment of non-infected wounds

AMPs provide different influences on the inflammatory phase by enhancing or decreasing the immune response. While tylotoin, a peptide from salamander skin with no antimicrobial activity, increased recruitment of macrophages to the wound site and promoted transforming growth factor (TGF)-β release from macrophages (Mu et al., 2014), the peptide CW49 from frog skin reduced macrophage recruitment and secretion of pro-inflammatory cytokines and might thus be used for the treatment of non-healing wounds associated with excessive inflammation (Liu et al., 2014a). Also the designer peptide tiger17 recruited macrophages to the wound site during the inflammatory phase and promoted TGF-β and interleukin (IL)-6 release from macrophages via activation of c-Jun N-terminal kinase (JNK) and extracellular signal-regulated kinase (ERK) mitogen activated protein (MAP) kinase pathways (Tang et al., 2014). AH90, a peptide from frog skin, induced wound healing by promoting TGF-β release from macrophages via activation of nuclear factor-κB (NF-κB) and JNK signaling pathways (Liu et al., 2014b).

**Figure 4:**
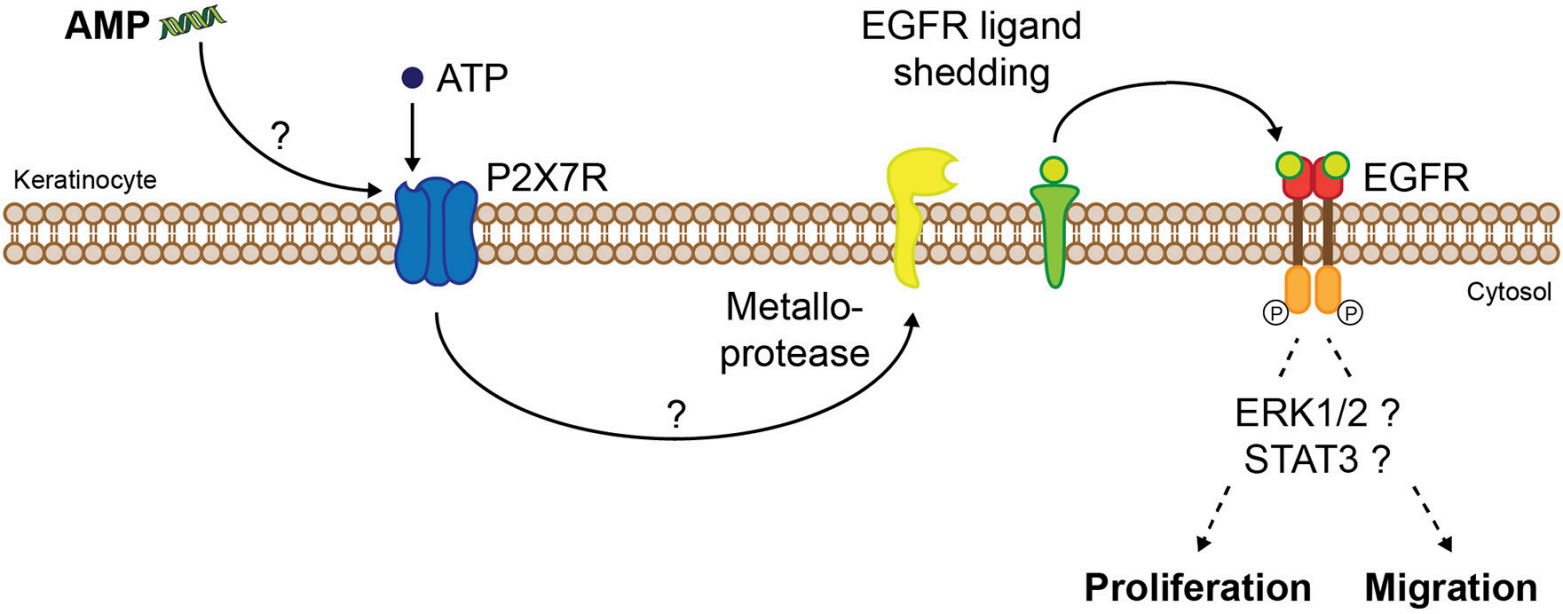
Proposed molecular mechanism of AMP-induced keratinocyte migration and/or proliferation via P2X7R and EGFR. AMPs induce P2X7R activation indirectly or by acting as allosteric modulators, thus increasing sensitivity of the extracellular ligand adenosine-triphosphate (ATP). P2X7R activation leads to EGFR transactivation via metalloprotease-mediated shedding of EGFR ligands which after cleavage from their membrane-anchored form trigger EGFR signaling. Activation of distinct signaling pathways finally leads to migration and/or proliferation of keratinocytes.

A number of peptides act via epidermal growth factor receptor (EGFR) transactivation and thus promote cell migration, a pivotal step during re-epithelialization, which is essential for adequate wound closure. This mechanism has been demonstrated for naturally occurring AMPs such as LL-37 (Tokumaru et al., 2005), melittin (Sommer et al., 2012), and SPINK9 (Sperrhacke et al., 2014), the frog-skin derived peptide esculentin-1a(1-21)NH_2_ (Di Grazia et al., 2015b), but also for the synthetic peptides Pep19-2.5 and Pep19-4LF (Pfalzgraff et al., 2016), DRGN-1 (Chung et al., 2017), and SHAP1 (Kim et al., 2014). EGFR transactivation is mediated via metalloprotease-dependent cleavage of membrane-bound EGFR ligands resulting in downstream activation of ERK1/2 or signal transducer and activator of transcription 3 pathway which ultimately promotes keratinocyte migration and/or proliferation (Figure 4). This effect seems to be stereospecific as an all-D-enantiomer of esculentin-1a(1-21)NH_2_ was not able to stimulate keratinocyte migration (Di Grazia et al., 2015b). Recent studies indicate that EGFR transactivation occurs via peptide-mediated activation of purinergic receptors, particularly the P2X7 receptor (P2X7R) seems to play a critical role (Figure 4; Sommer et al., 2012; Sperrhacke et al., 2014; Pfalzgraff et al., 2016; Comune et al., 2017). However, it is unclear whether the peptides indirectly activate the P2X7R or act as allosteric modulators similar to polymyxin B (Ferrari et al., 2004). For other AMPs the involvement of distinct receptors and pathways in cell migration and proliferation was demonstrated, such as the phosphoinositide 3-kinase (PI3K)/Akt/mechanistic target of rapamycin (mTOR) pathway (Tomioka et al., 2014) or G-protein coupled receptors, phospholipase C, EGFR, PI3K, ERK, and p38 MAPK (Hoq et al., 2011). Intriguingly, while some peptides solely influence cell migration but not proliferation (Carretero et al., 2008; Kim et al., 2014; Pfalzgraff et al., 2016), others are stimulating both (Niyonsaba et al., 2007; Sommer et al., 2012) despite their collective mode of action involving EGFR transactivation. Therefore, the EGFR downstream signaling pathways linked to peptide-induced cell migration and proliferation need to be explored further.

*In vivo*, AMPs strongly accelerate wound healing via induction of re-epithelialization and granulation tissue formation (Heilborn et al., 2003; Mu et al., 2014; Tang et al., 2014). The formation of new blood vessels which also belongs to the second wound healing stage can be supported by AMPs as well. Tylotoin promoted angiogenesis by inducing endothelial cell tube formation (Mu et al., 2014), while CW49 up-regulated angiogenic proteins (Liu et al., 2014a), WRL3 increased vascular endothelial growth factor production (Ma et al., 2017), and LL-37 mediated angiogenesis via formyl peptide receptor-like 1 (Koczulla et al., 2003).

Also the third wound healing stage can be supported by AMPs. SR-0379 promoted contraction capacity of human dermal fibroblast cells and accelerated *in vivo* wound healing by increasing collagen production (Tomioka et al., 2014) and Epi-1 enhanced the formation of collagen around the wound region (Huang et al., 2017). Other peptides can induce fibroblast-to-myofibroblast differentiation (Liu et al., 2014b; Mu et al., 2014; Tang et al., 2014) which synthesize extracellular matrix components such as collagen (Gabbiani, 2003). Further, α-smooth muscle actin expression can be increased in fibroblasts, therefore inducing wound contraction (Park et al., 2017).

### Treatment of SSTIs and infected wounds

Since skin barrier disruption is accompanied with high risk of infections and bacteria like *S. aureus* and *P. aeruginosa* prolong the inflammatory phase of wound healing, AMPs might facilitate wound recovery by combating the concomitant infection. The AMP proline-novispirin G10 (P-novispirin G10) strongly reduced bacterial counts within 3 days in a porcine infected wound chamber model using *S. aureus*. The rapid activity of the peptide led to killing of bacteria before peptide degradation and inactivation (Jacobsen et al., 2007). PXL150 showed rapid and strong antimicrobial effects by changing the membrane potential of *S. aureus* in an *in vivo* model of full-thickness wounds in rats infected with MRSA and in an *ex vivo* model of pig skin infected with *S. aureus* (Myhrman et al., 2013). Formulated in hydroxypropyl cellulose gel PXL150 rapidly reduced bacterial counts in an *in vivo* mouse model of *P. aeruginosa* infected burn wounds (Björn et al., 2015). In a mouse wound infection model, K1K8 was as effective as mupirocin ointment in reducing MRSA bacteria counts in the wound with rapid killing and did not induce resistance after 15 passages of *S. aureus* while showing low hemolytic activity and high serum stability (Li Z. et al., 2016). A distinct mechanism was found for peptides derived from tetraspanin CD9 which blocked the adherence of *S. aureus* to keratinocytes, thus inhibiting or reducing invasion of the skin with this pathogen (Ventress et al., 2016).

Certain natural AMPs show strong antimicrobial activity *in vitro* which is antagonized under physiological conditions such as high salt concentrations or the presence of polyanionic polymers like glucoseaminoglycans or mucin (Fjell et al., 2011). Despite, they are still able to kill bacteria *in vivo* suggesting an additional mechanism besides the direct antimicrobial effect. The lactoferrin-derived AMP HLR1r decreased bacteria survival in an *ex vivo* skin model infected with *S. aureus* although showing a reduced *in vitro* efficacy in a wound-like environment (Björn et al., 2016). Furthermore, some peptides protect against bacterial infections *in vivo* without possessing direct antimicrobial activity (Scott et al., 2007; Nijnik et al., 2010). The immunomodulatory properties displayed by these peptides resulted in their depiction as HDPs.

*S. aureus* skin infections are often associated with excessive inflammation increasing the clinical severity of the infection which is more central for the clinical severity of the skin infection than the bacterial burden (Montgomery et al., 2013). Therefore, anti-inflammatory properties of AMPs might be beneficial particularly for the healing of chronic and non-healing wounds such as diabetic leg ulcers. FI-PRPRPL-5 enhanced wound closure compared to the antibiotic mupirocin in a mouse model of MRSA skin infection and also reduced the mean bacterial count, albeit with a less pronounced effect than the antibiotic (Thangamani et al., 2015). In contrast, the peptide was capable of reducing the levels of the pro-inflammatory cytokines TNF, IL-1β, and IL-6, while mupirocin only reduced IL-6 levels. Ca-Tx-II inhibited NF-κB activation and secretion of pro-inflammatory cytokines in the wound tissue of *S. aureus*-infected wounds, while cytokines and chemokines such as MCP-1, that play a key role for wound healing, were increased (Samy et al., 2014). Topical application of the 12-mer peptide WR12 or the 8-mer peptide D-IK8 strongly reduced bacterial counts in a mouse model of MRSA skin infection comparable to the topical application of fusidic acid or the oral application of linezolid (Mohamed et al., 2016). Furthermore, the peptides decreased TNF and IL-6 secretion, thus exhibiting an anti-inflammatory effect additionally to the antimicrobial activity. IDR1018 accelerated wound healing in *S. aureus*-infected porcine wounds and non-diabetic, but not in diabetic mouse wounds by increasing re-epithelialization and showed a stronger wound closure effect than LL-37 and HB-107 without displaying an antibacterial effect (Steinstraesser et al., 2012). The authors postulated that the lack of effect in diabetic wounds might result from suppression of the wound healing promoting mechanism of the peptide in diabetic wounds due to a compromised immune system.

The innate defense-regulator peptide (IDR-1) modified signaling pathways downstream of TLRs and upregulated chemokines that recruit monocytes and macrophages to the site of infection, but downregulated pro-inflammatory cytokines (Scott et al., 2007). The cathelicidin HDP fowl-1 (6-26) protected mice from MRSA infection by enhancing the host response via increased chemotaxis and activation of macrophages and neutrophils (Bommineni et al., 2014). These peptides show the advantage of selectively enhancing host immune responses supporting the clearance of infection while on the contrary preventing overactivation of the immune system (Scott et al., 2007). Epi-1 recruited neutrophils to the wound site and decreased MRSA-induced inflammation and sepsis in heat wound-injured pigs, reflected in reduced IL-6 and C-reactive protein serum levels (Huang et al., 2017). For some immunomodulatory activities cell penetration of AMPs is required and can therefore particularly be provided by peptides that translocate not only into bacterial, but also into host cells (Mansour et al., 2015).

Activation or regulation of the immune system by AMPs is mainly determined by the cell type, the presence of other pro-inflammatory stimuli and the kinetics of the inflammatory response (Dutta and Das, 2015; Hemshekhar et al., 2016; Pinheiro da Silva and Machado, 2017). The dual ability of AMPs to mediate both pro- and anti-inflammatory responses likely takes place consecutively after an infection starting with the pro-inflammatory response which is activating the host immune response and thus facilitates the resolution of infection followed by the anti-inflammatory effect to avoid excessive inflammation. Regulation of inflammatory responses can also be determined by the local milieu, as exemplified by IDR-1018 which could increase MCP-1 production in macrophages in the absence of pro-inflammatory stimuli, while LPS-mediated cytokine production was reduced by the peptide (Choe et al., 2015). However, the most important role of AMPs seems to be selective control of pathogen-induced inflammation (Hemshekhar et al., 2016). Additionally, HDPs might be pro-inflammatory at high concentrations, while anti-inflammatory at low concentrations. hBD3 was highly expressed directly after an infection exerting a pro-inflammatory response, while thereafter the concentration decreased switching to an anti-inflammatory response to resolve inflammation (Semple et al., 2011). Several additional immunomodulatory mechanisms were found for AMPs where interaction with receptors is involved, including influence on dendritic cell maturation (Davidson et al., 2004; Kang et al., 2005) via G-protein coupled receptors and activation of the inflammasome and thus IL-1β secretion via the P2X7R (Ferrari et al., 2004; Tomasinsig et al., 2008), while melittin was capable of triggering IL-1β and IL-18 secretion in keratinocytes via the AIM2 inflammasome (Dombrowski et al., 2012). IL-1β secretion on the other side is involved in different stages of the wound healing process. LL-37 and IL-1β acted in synergy to promote angiogenesis (Salzer et al., 2014) and IL-1β could promote cell migration via EGFR (Sanchez-Guerrero et al., 2012). IDR-1018, cathelicidin-WA, and LL-37 influenced macrophage differentiation (van der Does et al., 2010; Chen et al., 2018). IDR-1018 provoked macrophage differentiation to an intermediate M1–M2 state with upregulation of M2 markers such as anti-inflammatory cytokines, but on the other side the opportunity to switch back to pro-inflammatory M1 macrophages and thus being able to respond adequately to infection (Mansour et al., 2015). As M2 macrophages are essentially involved in early and middle stages of wound repair, regulation of their differentiation by AMPs might contribute to improved wound healing (Ferrante and Leibovich, 2012).

Immunomodulatory effects of AMPs were also demonstrated in keratinocytes. Reduction of *IL-8* and cyclooxygenase (*COX)-2* gene expression in LPS-treated HaCaT keratinocytes was observed with a cryptic cationic AMP from human apolipoprotein E, probably due to direct binding to LPS (Pane et al., 2016). Similarly, the synthetic anti-endotoxin peptides Pep19-2.5 and Pep19-4LF which bind to and neutralize bacterial pathogenicity factors were able to reduce cytokine secretion in lipopeptide-stimulated keratinocytes (Pfalzgraff et al., 2016) and non-canonical inflammasome activation induced by cytoplasmic LPS (Pfalzgraff et al., 2017). Since keratinocytes are an important source of pro-inflammatory cytokines in skin inflammation and infection, the anti-inflammatory activity of AMPs in these cells might essentially contribute to the protection against complicated SSTIs. In addition, LL-37 protected keratinocytes from apoptosis mediated via a COX-2-dependent mechanism (Chamorro et al., 2009) and prolonged the life span of neutrophils, by suppressing apoptosis via formyl peptide receptor-like 1 and P2X7R (Nagaoka et al., 2006).

*C. (formerly P.) acnes* which is the second major Gram-positive pathogen in SSTIs (Ozolins et al., 2005) and shows increasing resistance to standard antibiotic therapies, is capable of triggering TLR2-mediated inflammation in acne lesions (Kim et al., 2002; Su et al., 2017). Current standard treatments are accompanied by severe side effects and low efficiency in many acne patients (Ryu et al., 2015). Thus, *C. acnes* represents an excellent target for the treatment with antimicrobial peptides due to their dual-immunomodulatory and antimicrobial activity and low resistance rate (Harder et al., 2013; Lim et al., 2015). AMPs were found to inhibit growth of *C. acnes* and release of pro-inflammatory cytokines in peripheral blood mononuclear cells while increasing anti-inflammatory cytokines (Popovic et al., 2012). Granulysin-derived peptides were able to reduce the clinical grade of acne when used in 30 patients as topical formulation for 12 weeks (Lim et al., 2015). Additionally, they showed bactericidal and anti-inflammatory activity *in vitro* against *C. acnes* (McInturff et al., 2005). The synthetic cecropin A–magainin 2 hybrid analog P5 was able to bind to lipoteichoic acid, thereby reducing *C. acnes*-induced TLR2 up-regulation and NF-κB nuclear translocation in keratinocytes, and to inhibit growth of *C. acnes* stronger than benzoyl peroxide. *In vivo*, an anti-inflammatory and antibacterial effect against *C. acnes* could be demonstrated after intradermal injection of P5 following *C. acnes* inoculation into mice ears (Ryu et al., 2015). Also melittin reduced *C. acnes*-induced inflammatory responses *in vitro* and *in vivo* by down-regulating pro-inflammatory cytokines and TLR2, thereby mediating its anti-inflammatory effect via NF-κB signaling (Lee et al., 2014).

As described above, the diverse immunomodulatory activities exhibited by HDPs underline the potential of AMPs for the therapeutical use in SSTIs and wounds. Specifically, AMPs possess various features beneficial for wound healing, including their direct antimicrobial effect which prevents infection that would otherwise delay wound healing. Wound recovery is further supported by immunomodulatory activities such as chemotactic activity toward macrophages and neutrophils, neutralization of bacterial pathogenicity factors and modulation of cytokine release which reduce detrimental pro-inflammatory responses. For the treatment of non-infected wounds, promotion of cell migration and proliferation, induction of angiogenesis, stimulation of collagen synthesis and wound contraction are especially favorable. However, it should be noted that many *in vivo* wound healing studies with AMPs were conducted in mice where wound healing mainly occurs via contraction and not re-epithelialization and granulation tissue formation (Wong et al., 2011). Thus, alternative preclinical models should be considered for *in vivo* wound healing experiments, as already realized for a number of AMPs that could show high activity in pig wound healing models (Ansell et al., 2012).

### Treatment of biofilms

A major issue in the treatment of SSTIs and wounds, but also in implants, are bacterial biofilms, a structured, surface-associated assemblage of microorganisms enclosed in an extracellular biopolymeric matrix. Compared to planktonic (free swimming) cells, bacteria growing in biofilms are 10- to 1,000-fold more resistant to conventional antibiotics, mostly due to limited penetration of antibiotics, and are estimated to account for at least 65% of all human infections and often result in chronification of infections and wounds. Biofilms are particularly difficult to eradicate due to their resistance to exogenous stresses such as therapy with conventional antibiotics and host defense mechanisms like opsonisation and phagocytosis (Luca et al., 2013; de la Fuente-Núñez et al., 2015). The biofilm constitutes a permeability barrier for antibiotics and the bacterial population within the biofilm is very heterogenic with a mixture of metabolically active and inactive cells (Stewart and Costerton, 2001; Batoni et al., 2011). *In vitro* and *in vivo* biofilm formation was demonstrated for pathogens involved in skin diseases such as *S. aureus* and *Streptococcus pyogenes* (Percival et al., 2012), but also *P. aeruginosa*, which when colonizing wounds, existed as biofilms rather than single cells (Kirketerp-Moller et al., 2008). *S. aureus* biofilms were shown to be more difficult to eradicate than their planktonic counterparts and may play an important role in acute and chronic cutaneous wound infections that are refractory to therapy (Davis et al., 2008). *S. aureus* and *S. epidermidis* biofilms formed in a mouse wound model caused disruption of normal re-epithelialization (Schierle et al., 2009). Additionally, the occurrence of biofilms is much greater in chronic compared to acute wounds (James et al., 2008). Thus, therapies that try to eradicate biofilms might improve recovery and healing of infected wounds.

IDR-1018 possesses a broad-spectrum anti-biofilm activity due to inhibiting a common stress response in bacteria (de la Fuente-Nunez et al., 2014). It promoted degradation of the widespread signaling nucleotide guanosine penta- and tetra-phosphate (p)ppGpp, an important signal in biofilm development and stress responses, and synergistically interacted with distinct antibiotics to prevent and eradicate bacterial biofilms (Reffuveille et al., 2014). The broad-spectrum anti-biofilm activity seems to originate from its prevention of (p)ppGpp-induced signaling effects. Other AMPs such as LL-37, which are able to prevent biofilm formation, inhibited initial bacteria attachment and quorum sensing systems and stimulated twitching motility and thus did not show a broad-spectrum anti-biofilm activity (Overhage et al., 2008). While 1,037, a synthetic, 9-amino-acid peptide, with very weak antimicrobial activity, inhibited biofilm formation of Gram-negative and Gram-positive bacteria also by stimulating twitching motility, it additionally reduced flagella-dependent swimming motility and bacterial swarming, and downregulated genes involved in biofilm formation including the quorum-sensing-regulated gene rhlB (de la Fuente-Nunez et al., 2012). A synthetic helical cathelicidin derived from NA-CATH, identified in a Chinese cobra, inhibited *S. aureus* biofilm formation also in the presence of salt with a stronger activity than NA-CATH and LL-37 and D-LL-37, thereby not affecting bacterial attachment, while LL-37 and D-LL37 reduced attachment of *S. aureus*. Interestingly, the anti-biofilm activity of all these peptides was salt-independent, while they lost their antimicrobial activity against planktonic bacteria (Dean et al., 2011a). In contrast, synthetic peptide variations of NA-CATH did not inhibit biofilm formation of *P. aeruginosa* (Dean et al., 2011b). The unnatural, proline rich AMP FI-P_R_P_R_P_L_-5 was able to reduce biofilm mass from preformed biofilms generated by *S. aureus* strains stronger than mupirocin and vancomycin (Thangamani et al., 2015). Also lactoferrin increased twitching motility of *P. aeruginosa* by decreasing free iron levels due to chelating iron and thus is able to block biofilm development (Singh et al., 2002). In combination with xylitol, lactoferrin could inhibit growth of established biofilms of a clinical wound isolate from *P. aeruginosa* and a lactoferrin/xylitol hydrogel in combination with silver wound dressings showed a stronger biofilm viability reduction than a commercially available wound hydrogel in *P. aeruginosa* and MRSA (Ammons et al., 2011).

Acylation of AMPs can have an influence on the bactericidal effect against planktonic and biofilm bacteria. In synthetic peptides, based on LF-11, an 11-mer peptide derived from human lactoferricin, peptide acylation increased the antimicrobial activity against planktonic bacteria (Sanchez-Gomez et al., 2015). Hydrophobicity, however, reduced anti-biofilm activity, probably due to interaction with exopolysaccharide components of the biofilm resulting in aggregation and thus reduced penetration, as suggested by the authors. In contrast, for cyclic lipopeptides derived from fusaricidin/LI-F class of AMPs, an increase of overall hydrophobicity and net positive charge improved activity against biofilms due to an improved biofilm penetration (Bionda et al., 2016). These peptides, like peptides targeting (p)ppGpp, also showed a broad-spectrum anti-biofilm activity.

Another mechanism of action for anti-biofilm activity is displayed by the amphibian skin peptide esculentin (1–21) which disrupted biofilms by bacterial membrane perturbation of *P. aeruginosa* with increased leakage of the cytosolic enzyme β-galactosidase (Luca et al., 2013). The same mode of action was exhibited by the peptide against free-living forms of *P. aeruginosa*. Since the effect occurred within 15 min intracellular processes can be excluded. Bacteriocins caused formation of stable pores on biofilm cells which resulted in ATP efflux and consequently cell death. For the two bacteriocins nisin A and lacticin Q penetration of the biofilm matrix could be demonstrated reaching the deepest part of the biofilm (Okuda et al., 2013). Also the small lytic peptide PTP-7 was able to diffuse into deep layers of *S. aureus* biofilms and kill bacteria inside the biofilm by causing cell lysis (Kharidia and Liang, 2011). RRIKA and RR were able to disperse mature *S. aureus* and *S. epidermidis* biofilms with a stronger biofilm reduction than the antibiotics vancomycin and linezolid (Mohamed et al., 2014). Further AMPs with specific anti-biofilm activity are the tryptophane-rich antibacterial peptides KT2 and RT2 preventing *E. coli* biofilm formation and killing bacteria in mature *E. coli* biofilms by translocating across the bacterial membrane after binding to bacterial surface LPS and binding to DNA after entry into the bacterial cells (Anunthawan et al., 2015). Stereospecificity seems to play an important role for anti-biofilm activity as IK8L, a short synthetic peptide with all L-amino acids (AA) was more active against MDR *P. aeruginosa* biofilms than the same peptide with 4 D-AA or an all D-AA peptide (Zhong et al., 2017). Furthermore, it was superior to the antibiotic ceftazidime with more rapid bactericidal activity *in vitro* with the advantage of negligible resistance and superior to imipinem as topical application *in vivo* in an infected mouse burn wound model by increasing survival and reducing bacterial counts. The synthetic and anti-biofilm peptide SAAP-148 was applied as a single 4-h treatment in hypromellose ointment and completely eradicated acute and established, biofilm-associated infections with MRSA and MDR *A. baumannii* from wounded *ex vivo* human skin and mouse skin *in vivo* (de Breij et al., 2018). Cyclic lipopeptides showed strong activity against staphylococcal biofilms in a porcine wound model (Bionda et al., 2014) and DJK-5 and DJK-6 appear to be promising candidates to treat biofilms *in vivo* since they protected the invertebrate organisms *Caenorhabditis elegans* and *Galleria mellonella* from otherwise lethal *P. aeruginosa* biofilm infections (de la Fuente-Núñez et al., 2015). DRGN-1 was able to promote wound healing in mixed biofilm-infected wounds by inducing the clearance of *S. aureus* and *P. aeruginosa* biofilms combined with increased keratinocyte migration (Chung et al., 2017). Importantly, the AMPs tested for anti-biofilm activity *in vivo* showed negligible toxicity (de la Fuente-Núñez et al., 2015; Zhong et al., 2017; de Breij et al., 2018).

It is evident from the above studies that AMPs as anti-biofilm agents possess advantageous features such as their broad-spectrum, rapid activity, low susceptibility to resistance development and a bactericidal effect independent of the bacterial growing state, whereas conventional antibiotics kill actively dividing cells.

## Strategies to improve topical bioavailability of AMPs

In general, topical therapy is preferred over systemic drug administration in skin diseases to avoid systemic adverse effects. However, topical application requires sufficient tissue penetration (Stein and Wells, 2010). The skin, especially the stratum corneum, represents an effective barrier for topical treatment due to highly organized stratum corneum lipids, thus impeding the penetration of peptides into the skin. Additionally, rapid degradation e.g., by aminopeptidases and esterases might limit the topical use of AMPs, particularly for large molecules such as melittin, a 26-amino acid peptide, but also for hydrophilic and very lipophilic molecules. However, skin penetration of AMPs like melittin might be facilitated by their membrane-disrupting mode of action and their cytotoxic effect. For some AMPs penetration in deeper layers of the skin was demonstrated revealing slow access to the viable skin after 24 h (Do et al., 2014). Another major issue for the clinical use of AMPs is their low stability *in vivo*, e.g., toward bacterial proteases, but also to endogenous proteases such as trypsin-like proteases that are abundant in the body (e.g., in wound exsudate) and selective for basic residues. The majority of naturally occurring AMPs have unfavorable pharmacokinetic properties such as a very short half-life of only 1–2 h, mainly due to degradation by proteases (Di Grazia et al., 2015a). Thus, if intended for clinical use, peptides should be examined for skin penetration and effects of skin enzymes on peptide activity.

**Figure 5:**
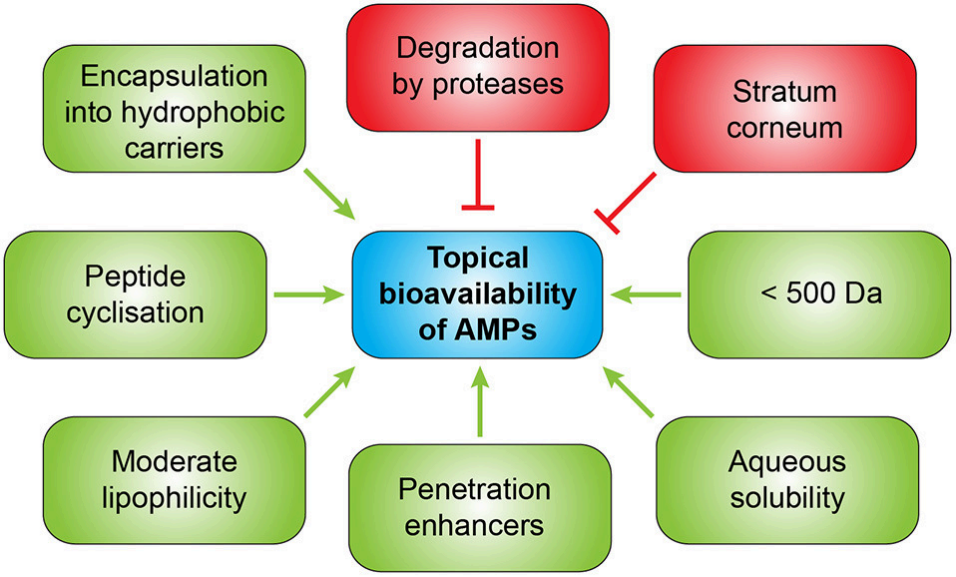
Factors most likely determining the extent of topical bioavailability of AMPs.

Several strategies exist to improve the bioavailability of AMPs for topical application (Figure 5). To enhance penetration different approaches can be used such as administration together with penetration enhancers, encapsulation into hydrophobic carriers or chemical modifications of the peptides to increase lipophilicity (Namjoshi and Benson, 2010). A combination of an enzyme which inhibits biofilm formation and disperses pre-formed biofilms with an anti-biofilm peptide in a gel formulation showed synergistic effects against chronic wound infections (Gawande et al., 2014). Since drug permeation through the stratum corneum is widely dependent on molecular size and lipophilicity, smaller AMPs display the advantage of improved penetration. A molecular weight lower than 500 Da (Bos and Meinardi, 2000) and moderate lipophilicity and aqueous solubility are ideal characteristics for successful transdermal delivery. Furthermore, antimicrobial peptides used for topical application should not be absorbed from the wound or infection site into the systemic circulation and not provoke allergic sensitization which could be demonstrated for pexiganan which already passed through clinical trials for the treatment of mildly infected, diabetic foot ulcers, but was not approved by the FDA despite being clinical comparable to the oral antibiotic ofloxacin (Lipsky et al., 2008). Skin penetration of AMPs might be improved by cyclization which improves their chemical and enzymatic stability, receptor selectivity, and pharmacodynamic properties (Namjoshi and Benson, 2010). An alternative therapeutic option could be the induction of endogenous AMPs as demonstrated for aroylated phenylenediamines which could induce LL-37 up to 20- to 30-fold *in vitro* (Ottosson et al., 2016).

Different approaches were made to improve the stability of AMPs such as introduction of D-AA (Gao et al., 2016). For cationic α-helical peptides a minimum ratio of D-AA of 33% was shown to be required for complete resistance to enzymatic degradation (Lee et al., 2005; Wang et al., 2010). D-AA further prohibit aggregation of AMPs and reduce toxicity due to destabilized helical structures (Li H. et al., 2016). Likewise, modifications of the terminal regions such as acetylation, amidation and hydrophobic tagging and the use of peptidomimetics reduce susceptibility to proteases (Ebbensgaard et al., 2015; Ageitos et al., 2017). Another possibility is substitution with the amino acid tryptophan which could reduce proteolytic degradation of the LL-37-derived peptide EFK17 and improved its antimicrobial potency (Stromstedt et al., 2009). WR12 which is exclusively composed of Trp and Arg residues showed an improved antimicrobial salt stability compared to pexiganan (Mohamed et al., 2016). Likewise pegylation can increase resistance to bacterial proteases (Falciani et al., 2014).

Another promising approach seems to be the coating of biomaterials like biomedical devices and implants with AMPs which showed encouraging effects *in vitro* (Kazemzadeh-Narbat et al., 2010; Deslouches et al., 2015; Kasetty et al., 2015; Yazici et al., 2016) and *in vivo* (Choe et al., 2015; Chen et al., 2016), hence reducing bacterial colonization and allowing controlled release of the peptides with high concentrations for prolonged periods. For this purpose the peptides can be incorporated in distinct matrices such as a polymer-lipid encapsulation matrix (de Breij et al., 2016).

Salt resistance and serum stability can be improved by insertion of the non-natural amino acid β-naphtlylalanine or replacement of tryptophans and histidines in Trp- and His-rich AMPs with this bulky amino acid, which on the other side increases hemolytic activity (Chu et al., 2013). Also tryptophane end tagging can improve salt stability with the advantage of lower hemolytic activity compared to β-naphtlylalanine.

To improve the bioavailability of AMPs several drug delivery systems such as hydrogels (Xie et al., 2015), poly(lactic-co-glycolic acid) (PLGA) nanoparticles, or lipid-based peptide encapsulation systems (MeIkle et al., 2017) with incorporated AMPs were investigated with sustained delivery of the peptides and controlled release. Furthermore, delivery systems can improve solubility and provide targeted delivery. Esculentin-1a(1-21)NH_2_ coated on gold-nanoparticles (AuNPs) showed increased activity against *P. aeruginosa* and resistance to proteolytic digestion compared to the free peptide (Casciaro et al., 2017). The advantage for topical therapy using AuNPs is their ability to penetrate all layers of human skin to a greater extent than other metal nanoparticles such as silver NPs. LL-37-conjugated gold nanoparticles showed higher *in vivo* wound healing activity compared to LL-37 alone and prolonged phosphorylation of EGFR and ERK1/2 *in vitro*, thus improving cell migration (Comune et al., 2017). Encapsulation of solid lipid nanoparticles (SLNs) with LL-37 in the combination with the elastase inhibitor Serpin A1 provided a biphasic release profile with strong release at the beginning and sustained release afterwards over the entire study period of 15 days while permeation of LL-37 alone discontinued after 2 days (Fumakia and Ho, 2016). This formulation was protecting the peptide from degradation and could permeate across the skin, while accelerating wound healing *in vitro* and showing synergistic effects against *S. aureus* and *E. coli* compared to the single drug. Coating of various surfaces with battacin lipopeptides prevented colonization by *P. aeruginosa* and *E. coli* biofilms and might thus be used for preventing colonization of medical implants (De Zoysa and Sarojini, 2017). The importance of the formulation was further demonstrated for the AMP P60.4Ac which was investigated in two different cream formulations, PBS or a hypromellose gel (Haisma et al., 2016). In the gel formulation P60.4Ac was most effective against *S. aureus*, including MRSA and mupirocin-resistant MRSA, while its antimicrobial activity was reduced in the cream formulations and in PBS. While the peptide seemed to be released from the gel in adequate amounts within a longer time interval, release from cream formulations appeared insufficient. A3-APO and its monomeric metabolite were applied topical or i.m. revealing the same effectiveness in mouse models of intradermal infection of a burn wound with MRSA or intradermal infection with *C. acnes* (Ostorhazi et al., 2013). Moreover, when APO was incorporated into polyvinyl alcohol nanofiber which was polymerized into a solid patch dressing, it enhanced wound healing and reduced bacterial load superior to the antibiotic colistin in *Acinetobacter baumanii*-infected skin wounds in mice. The antibacterial activity *in vivo* of the peptide-loaded patch dressing was exceeding the effect of i.m. administration of the peptide without the dressing, probably due to a controlled release from the wound dressing (Sebe et al., 2016). For medical application wound dressings seem to be a promising option as demonstrated for cotton gauze with Cys-LC-LL-37 or magainin 1 which blocked bacterial growth while showing very low cytotoxicity (Gomes et al., 2015). AMP encapsulation in a thermosensitive hydrogel or incorporation of peptides conjugated to dextrin into hydrogels has been described (Li et al., 2015; Silva et al., 2015). While improving bioavailability, the local delivery system can also serve as drug depot with sustained release of the peptides and leading to a higher local drug concentration. These delivery systems should be biocompatible, non-immunogenic, and biodegradable (Chereddy et al., 2014).

## Perspectives for future therapy

Antibiotics have the major disadvantage to cause release of PAMPs from pathogens resulting in damaging inflammation and sepsis. This especially applies to antibiotics targeting the bacterial cell wall (Brandenburg et al., 2016). Hence, combination therapy of antibiotics together with antimicrobial peptides seems to be a promising approach and might prevent the development of bacterial resistance and reduce the drug dose required. Especially dose reduction is an important factor since this can reduce side effects which in turn play a critical role for clinical development.

AMPs can further act as antiresistance compounds for conventional antibiotics, e.g., via facilitating the access of antibiotics into bacterial cells by increasing the permeability of the cytoplasmic membrane (Mataraci and Dosler, 2012). Azithromycin acted in synergy with LL-37 and colistin which increased the permeability of the outer membrane of Gram-negative bacteria and thus facilitated the entry of azithromycin (Lin et al., 2015). The synthetic AMPs RRIKA and RR showed synergistic activity with lysostaphin against MSSA, MRSA, and VRSA isolates (Mohamed et al., 2014). The authors speculated that the synergistic effect might be due to lysostaphin-induced cleavage of peptidoglycan which is located in the cell wall allowing a better access of the peptides to the cell membrane. Esculentin-1b(1-18)NH2 in subinhibitory concentrations exhibited synergism with erythromycin and other antibiotics since it was able to increase the membrane permeability of *E. coli*, thus enhancing the access of the antibiotic to its intracellular target (Marcellini et al., 2009). Additionally, synergism could be shown for the combination of two antimicrobial peptides (Capparelli et al., 2009; Rahnamaeian et al., 2015; Zhao et al., 2015; Field et al., 2016; Mohamed et al., 2016) whereas combination of three AMPs displayed an even stronger synergism (Yu et al., 2016). The fact that AMPs can synergize with other AMPs and not only with conventional antibiotics might be used to increase therapeutic efficacy. IDR-1018 and DJK-5 and DJK-6 were acting in synergy with conventional antibiotics to prevent biofilm formation and treat pre-existing biofilms which allowed the use of lower concentrations of the antibiotics (Reffuveille et al., 2014; de la Fuente-Núñez et al., 2015). The proposed mechanism for the synergism was explained by an increased bacterial susceptibility to antibiotics due to the capability of the peptides to target (p)ppGpp and to downregulate genes involved in the mechanism of action of antibiotics and biofilm formation. Furthermore, it could be demonstrated that AMPs are able to extend the spectrum of antibiotics. For the conjugation of the mouse peptide CRAMP and the antibiotic vancomycin, normally used for the treatment of infections with Gram-positive bacteria, activity against both Gram-positive and Gram-negative bacteria was shown, presumably due to translocation of vancomycin into the periplasm of Gram-negative bacteria enabled by the AMP (Pletzer et al., 2016). Additionally, AMPs are able to suppress resistance of bacteria to conventional antibiotics (Mohamed et al., 2016). Incubation with subinhibitory concentrations of the 12-mer peptide WR12 with VRSA strains re-sensitized them to the effect of vancomycin, teicoplanin, and oxacillin. The synthetic α-helical peptide PL-5 showed synergism with levofloxacin in a *S. aureus* mouse infection model *in vivo*, probably due to a dual mode of action with the peptide targeting the bacterial membrane and the antibiotic with cytoplasmic targets (Feng et al., 2015).

Thus, it would be expected that combination therapies will have several major advantages including broad spectrum of coverage, the ability to reduce concentrations of single drugs and thus their side effects and to delay drug resistance evolution.

## Conclusion and perspective

Enormous progress has been made in peptide development and in unveiling the various characteristics of AMPs. Favorable features for the treatment of skin infections and wounds are the efficacy against biofilms, the capability of promoting wound healing and the ability to modulate immunity by increasing protective immunity while on the other side dampening overwhelming inflammatory responses accompanied in part by direct antimicrobial effects.

The dual bioactivity displayed by AMPs, namely their propensity to control both infection and inflammation, might be beneficial particularly for the treatment of chronic wounds and complicated SSTIs associated with strong inflammatory responses. While supporting infection-resolving immunity and modulating innate immunity, they dampen potentially harmful pro-inflammatory responses. Nevertheless, their diverse functions in immunity elicited by binding to distinct receptors or influencing different intracellular signaling pathways still have to be elucidated to avoid occurrence of side effects that could emerge when using these highly potent molecules in therapy. Tight regulation of AMPs is required since high or persistent amounts might cause chronic inflammation as demonstrated for psoriasis and rosacea.

The knowledge regarding direct interaction between AMPs and endogenous receptor proteins is still very limited. The natural AMP LL-37 is highlighting the complexity of the interaction between AMPs and the host since it is interacting with at least 16 proteins and receptors, thereby influencing more than 1,000 secondary effector proteins (Hancock et al., 2016). However, more than two decades of work were required to decipher the complex mechanisms reflecting its interaction with the host since it was first identified in 1995 (Vandamme et al., 2012). It would be interesting to decipher the distinct activities of AMPs that support wound healing and to identify relevant peptide structures, to develop peptides that ideally possess several features that are favorable for wound healing. However, peptides that are activating growth factor receptors should be screened for their potential to induce tumorigenesis (Weber et al., 2009). Additionally, *in vivo* testing of AMPs is unconditionally required as isolated cell systems cannot reflect the complexity of the innate immune response (Easton et al., 2009).

Despite the great efforts that were made by several research groups to design new peptides with improved properties, only few AMPs have been introduced to the market or are in clinical trials (Greber and Dawgul, 2017; Kang et al., 2017). Nevertheless, most of the studies conducted concentrate on topical therapy for indications such as acne vulgaris, rosacea, or chronic wounds revealing the promising therapeutic applicability of AMPs for these medical indications.

## Author contributions

All authors listed have made a substantial, direct, and intellectual contribution to the work and approved it for publication.

### Conflict of interest statement

KB is CSO of Brandenburg Antiinfektiva GmbH. All other authors declare no competing interests.

## Footnotes

^1^WHO: Antimicrobial resistance: Global report on surveillance 2014. http://www.who.int/drugresistance/documents/surveillancereport/en/

^2^The Antimicrobial Peptide Database (APD). http://aps.unmc.edu/AP/

## Abbreviations

α-SMA: alpha-smooth muscle actin
AD: atopic dermatitis
AMP: antimicrobial peptide
ATP: adenosine-triphosphate
COX: cyclooxygenase
DC: dendritic cell
EGFR: epidermal growth factor receptor
ERK: extracellular signal-regulated kinase
ESKAPE: *Enterococcus faecium, Staphylococcus aureus, Klebsiella pneumoniae, Acinetobacter baumannii, Pseudomonas aeruginosa*, and *Enterobacter* species
FPRL: formyl peptide receptor-like
GAS: group A *streptococcus*
IFN: interferon
IL: interleukin
ILC: innate lymphoid cell
hBD: human β-defensin
HDP: host defense peptide
HNP: human neutrophil peptide
i.m.: intramuscular
JNK: c-Jun N-terminal kinase
KLK: kallikrein-related peptidase
LP: lipoproteins/-peptides
LPS: lipopolysaccharide
MAP: mitogen-activated protein
MCP: monocyte chemoattractant protein
mDC: myeloid dendritic cell
MRSA: methicillin-resistant *Staphylococcus aureus*
MSSA: methicillin-sensitive *Staphylococcus aureus*
mTOR: mechanistic target of rapamycin
NF-κB: nuclear factor-kappa B
NOD2: nucleotide-binding oligomerization domain 2
P2X7R: P2X7 receptor
PAMP: pathogen-associated molecular pattern
PBS: phosphate-buffered saline
pDC: plasmacytoid dendritic cell
PI3K: phosphoinositide 3-kinase
PLGA: poly (lactic-co-glycolic acid)
PSM: phenol-soluble modulin
RNS: reactive nitrogen species
ROS: reactive oxygen species
SSTI: skin and soft tissue infection
STAT: signal transducer and activator of transcription
TGF-β: transforming growth factor-beta
Th: helper T cell
TNF: tumor necrosis factor
TLR: Toll-like receptor
VD3: vitamin D3
VRE: vancomycin-resistant *Enterococcus*
VRSA: vancomycin-resistant *Staphylococcus aureus*.
